# Role of the Microbiota in Colorectal Cancer: Updates on Microbial Associations and Therapeutic Implications

**DOI:** 10.1089/biores.2016.0028

**Published:** 2016-10-01

**Authors:** Olivia I. Coleman, Tiago Nunes

**Affiliations:** Chair of Nutrition and Immunology, ZIEL—Research Center for Nutrition and Food Sciences, Technical University of Munich, Freising, Germany.

**Keywords:** colorectal cancer, intestinal microbiota, tumorigenesis

## Abstract

Genetic, environmental, and dietary factors have been found to influence the development and progression of colorectal cancer (CRC). More recently, accumulating evidence associates the intestinal microbiota with the initiation and progression of this disease. While studies have shown that individuals with CRC display alterations in gut bacterial composition, it remains somewhat unclear whether such differences drive cancer development or whether they are a response to tumorigenesis. In this review, the authors assess new evidence linking the community structure or specific bacterial factors of the intestinal microbiota to CRC development and progression, with insights into therapeutic implications.

## Introduction

Colorectal cancer (CRC) is one of the leading causes of death in the western society, being ranked third most lethal neoplasia in the United States in both men and women.^1^ In 2014, the American Cancer Society estimated that approximately 136,830 new cases of CRC will be diagnosed in the United States, with more than 50,000 Americans expected to die due to disease progression or complications.^1,2^ The lifetime cancer-related costs are considerable and differ by cancer site, disease stage, age at diagnosis, and treatment phase. Considering direct healthcare costs, CRC is the second most important neoplasia with estimated expenses of more than $14 billion.^3,4^

**FIG. 1.**
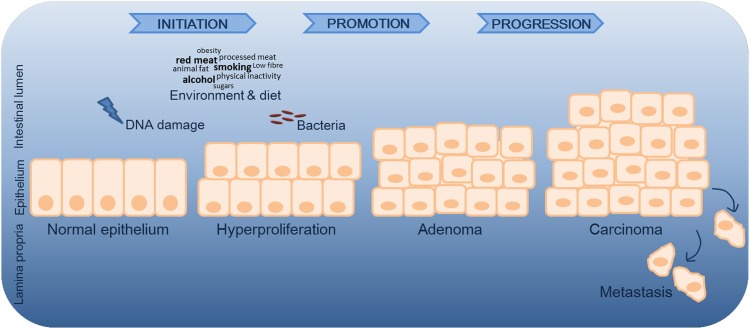
Simplified representation of CRC progression. In the initiation process, normal cells go through early unrepaired changes in DNA sequence and structure, which ultimately lead to their transformation into neoplastic cells.^6–8^ In the promotion phase, mutated cells undergo clonal expansion, promoting atypical tissue growth and tumor formation. In addition to genetic mutations, environmental and microbial factors contribute to disease progression. Microbes may contribute by either promoting or suppressing CRC development, with bacteria being described as drivers and/or passengers of disease.^58^ In the progression phase, malignant tumor transformation and expansion take place.^7^ CRC, colorectal cancer.

Most cases of CRC originate from epithelial cells of the colorectal mucosa, being identified by the formation of glandular structures and histologically classified as adenocarcinomas.^5^ The development of CRC can be didactically viewed as a systematic process with three main stages: initiation, promotion, and progression (Fig. 1).^6^ In the initiation process, either spontaneously or after exposure to carcinogenic initiators, normal cells go through early unrepaired changes in DNA sequence and structure, which ultimately lead to their transformation into neoplastic cells.^6–8^ In the promotion phase, mutated cells undergo clonal expansion, promoting atypical tissue growth and tumor formation. In the progression phase, malignant tumor transformation and expansion take place with the occurrence of additional mutations, epigenetic alterations, and genetic instability.^7^ Thus, CRC development results from a progressive loss of normal control mechanisms related to cellular growth and differentiation.

Owing to the fact that single mutations are not sufficient to trigger malignant transformation in the intestinal epithelium,^7^ an accumulation of multiple mutations in proto-oncogenes, tumor suppressor genes, and DNA repair genes is needed to complete the carcinogenesis process. Most genetic alterations are found in pathways related to Wnt-*β*-catenin signaling, tyrosine kinase receptors, TGF*β* signaling, DNA mismatch repair, and genes linked to apoptotic pathways and cell cycle control.^7^ In addition to genetic alterations, the tumor microenvironment plays a critical role in CRC initiation and promotion, with the dietary intake and the intestinal microbiota being the most dominant factors of the luminal microenvironment in the gut. It has therefore been suggested that differences in diet and in the intestinal microbiota might be accountable for variations in CRC prevalence between two similar human populations. As an example, CRC is extremely rare in Native Africans, but considerably prevalent in African Americans (<1 case per 100,000 population vs. 65 per 100,000 population).^9^ In these populations, O'Keefe et al. showed that a larger consumption of animal products and an increased colonic population of toxic hydrogen and secondary bile salt-producing bacteria among African Americans were associated with increased CRC rates, supporting the hypothesis that CRC risk is affected by the interplay between diet and the intestinal microbiota.^9^ This review discusses the current evidence covering the interactions between the intestinal microbiota and the host in the development and progression of CRC.

## Microbiota, Genotoxicity, and Immune Activation

Viral and bacterial infections are known to facilitate carcinogenesis in certain organs. Prominent examples include viral hepatitis and hepatocellular carcinoma,^10–12^ as well as *Helicobacter pylori* infection and gastric adenocarcinoma.^13–15^ In the case of the hepatitis B virus (HBV), for instance, the infection can contribute to liver carcinogenesis through direct and indirect mechanisms: genomic instability due to HBV-DNA integration into the host genome, deregulation of proliferation control by viral regulatory proteins, and epigenetic alterations driven by viral compounds targeting the expression of tumor suppressor genes.^11^ The secretion of virulence factors by *H. pylori* causes oxidative stress, chronic inflammation, and host DNA damage, resulting in carcinoma development.^16–18^ While there is a well-established link between inflammation, carcinogenesis, and microbial products, the function of the microbiota in initiating and promoting CRC is not well understood.^19^

**FIG. 2.**
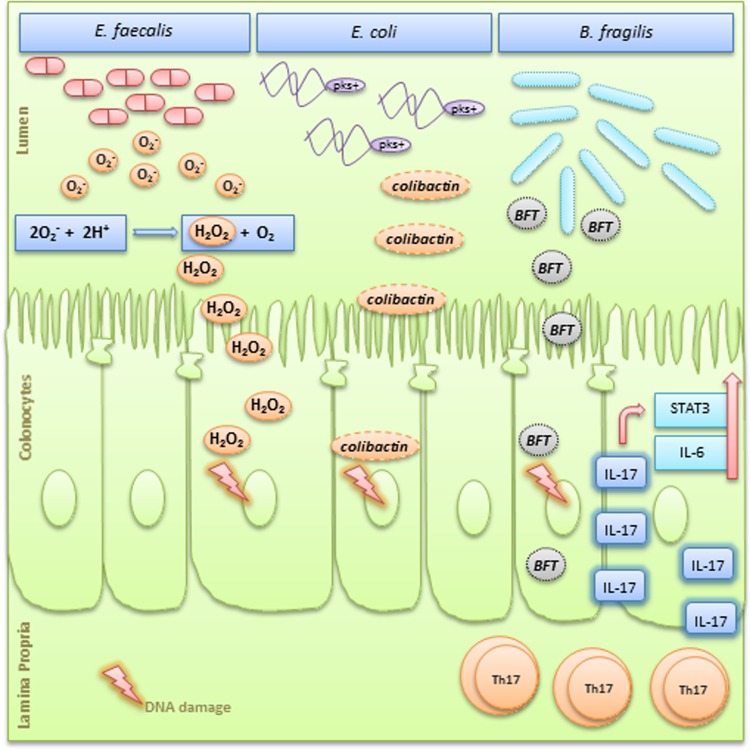
Simplified graphic showing three different microorganisms and their pro-tumorigenesis mechanisms. *Enterococcus faecalis* produces extracellular superoxide (O_2_^−^) near the oxygenated luminal surface of colonic epithelial cells. In this acidic microenvironment, O_2_^−^ production spontaneously generates H_2_O_2_ that diffuses into the epithelium and forms hydroxyl radicals at DNA sites, leading to DNA–protein crosslinks, DNA breaks, and base modifications.^41^ *Escherichia coli* of the phylogenetic group B2 carry a conserved genomic island named “pks,” which allows the production of a genotoxin named colibactin. Colibactin can induce DNA double-strand breaks leading to chromosomal aberrations and increased frequency of gene mutations.^22^ ETBF is a subtype characterized by the secretion of a metalloprotease enterotoxin known as BFT. BFT is directly genotoxic to colonic epithelial cells and also stimulates cleavage of E-cadherin causing cell proliferation and breakage of the intestinal barrier.^58^ BFT can also induce a persistent TH17-type inflammatory response with increased IL-17 expression and upregulation of STAT3 and IL-6, which have pro-proliferative and antiapoptotic properties.^58^ BFT, *Bacteroides fragilis* toxin; ETBF, enterotoxigenic *Bacteroides fragilis*.

For each cancer-associated infection, microorganisms can trigger common and etiology-specific carcinogenic pathways, having both direct and indirect neoplastic effects that go beyond the immune activation and the development of chronic inflammation (Fig. 2). *Enterococcus faecalis*, for instance, is known to produce extracellular superoxide that can induce chromosomal instability in human cells.^19,20^ In this regard, Wang et al. have shown that *E. faecalis* can activate DNA damage pathways, produce G2 arrest, and promote missegregation of chromosomes leading to aneuploidy and tetraploidy in colonic epithelial cells *in vitro*.^19^ *In vivo* studies have confirmed this potential neoplastic influence, demonstrating that gnotobiotic IL-10-deficient mice colonized with *E. faecalis*, developed colitis-associated rectal dysplasia and adenocarcinoma.^21^ Another example is the *Escherichia coli* of the phylogenetic group B2, which can produce the genotoxin named colibactin.^22,23^ Infection experiments with these strains induce DNA double-strand breaks in intestinal epithelial cells leading to mitotic and chromosomal aberrations together with an increased frequency of gene mutations and anchorage-independent growth.^22^ Importantly, Buc et al. showed a higher prevalence of colibactin-producing *E. coli* in biopsies of patients with CRC compared with those of patients with diverticulosis.^24^ An additional microorganism found to be associated with CRC is the Enterotoxigenic *Bacteroides fragilis* (ETBF), a subtype characterized by the secretion of a 20-kDa metalloprotease enterotoxin known as *B. fragilis* toxin (BFT).^25^ When intestinal epithelial cell lines are exposed to this enterotoxin, cell adhesion molecules are cleaved, stress response and cytokine signaling pathways are activated, and an increased cellular proliferation, mediated by elevated expression of the c-Myc oncogene, takes place.^25^

Away from direct genotoxic capabilities, bacteria can also participate in tumorigenesis by promoting chronic unresolved inflammation. In this regard, epithelial barrier disruption and subsequent immune recognition of bacterial factors can lead to inflammation-driven neoplastic formation.^26^ Different bacterial species can initiate immune-mediated inflammation with characteristic kinetics and anatomic distribution.^27^ The importance of bacteria in inflammation-driven tumorigenesis is stressed by the decreased tumor formation found in several CRC mouse models housed in germ-free conditions or under antibiotic treatment. Accordingly, the inhibition of microbial recognition through the loss of pattern recognition receptor signaling or T helper cell activation leads to a diminished neoplastic transformation.^26,28–30^ In this regard, the knockout of the adapter MyD88, which participates in the downstream signaling of toll-like receptors, was shown to inhibit tumorigenesis in both Apc^Min/+^ mice^29^ and azoxymethane (AOM)-2% dextran sodium sulfate (DSS)-induced models.^28,30^ Importantly, not only the absence of bacteria can lead to decreased neoplastic transformation, but superimposed colonic infection can also enhance intestinal tumorigenesis in Apc^Min/+^ mice.^31–33^

In animal models with colitis-associated CRC, the resulting inflammatory microenvironment leads to elevated levels of reactive oxygen species (ROS) and prolonged immune activation, which may result in tissue damage, stimulation of oncogenes, and downregulation of tumor suppressor genes.^25^ However, the exact mechanisms by which inflammation promotes carcinogenesis are still poorly understood. Animal studies support the relevance of nuclear factor-κB (NF-κB) signaling in inflammation-driven carcinogenesis and the importance of IL-6 in this context.^26,34–37^ IL-6 induces STAT3-mediated signal transduction affecting proliferative, antiapoptotic, and proangiogenic genes.^26,38–40^ Elevated levels of ROS might also play an important role in bacteria-driven carcinogenesis as enteric bacteria have been shown to induce ROS, and mice lacking enzymes that protect against free radicals, such as glutathione peroxidases Gpx-1 and Gpx-2, are more susceptible to intestinal inflammation and tumorigenesis.^25,33,41^ In the case of *H. pylori* and *B. fragilis* infection of stomach and intestine, respectively, the main source of ROS production is associated with the polyamine catabolic enzyme spermine oxidase (SMO) generating H_2_O_2_ from the conversion of spermine to spermidine.^25^ SMO is promptly induced by these bacteria leading to SMO-dependent ROS production and DNA damage.^25^

Chronic innate inflammatory responses are often associated with tumorigenesis while adaptive immunity might inhibit the process.^26^ T cells are often linked to antitumor responses as more colonic tumor development is increased in *Rag*−/− mice and in animal models with defective interferon signaling,^26,42–44^ supporting the hypothesis that T cell-driven immunity is linked to tumor protective responses. Lymphocyte-driven immune responses, although not absolutely required, have a critical role in regulating bacteria-induced intestinal inflammation and this inflammatory response may influence the progression of CRC.^42^ Erdman et al. showed that *Helicobacter hepaticus*-infected Rag-2-deficient mice developed colitis-associated carcinoma, whereas uninfected mice did not.^42^ In addition, adoptive transfer of CD4^+^ CD45RB^lo^ CD25^+^T cells significantly inhibited colitis and cancer in this model, indicating that lymphocytes may be able to inhibit bacteria-induced inflammation and tumor formation.^42^ Several studies, however, have shown that T helper cell subsets have a differential role in cancer development. In this context, TH1 immunity is involved in antineoplastic responses, whereas TH17 contributes to tumorigenic responses.^26,43,44^ In this regard, Wu et al. have shown that ETBF colonizes Apc^Min/+^ mice associated with the activation of STAT3 and TH17 responses leading to increased colonic tumor development in these animals.^26^

It is also important to point out that instead of having direct proneoplastic effects in the colon, chronic inflammation might work indirectly by targeting the intestinal microbiota to promote the expansion of microbes with genotoxic capacities.^23^ In line with this, Arthur et al. showed that inflamed IL-10-deficient mice exhibited a 100-fold increase in the *E. coli* community, and that colibactin-producing *E. coli* induced increased tumor multiplicity without altering the level of inflammation compared with nongenotoxic strains in monoassociation studies.^23^ These data suggest that inflammation *per se* might not be the main contributing factor in tumor formation and that inflammation-driven selection of genotoxic bacteria within the complex community of the intestinal microbiota may link colonic inflammation and CRC development.

## The Microbiota and CRC in Human Studies

Individuals with CRC display instability in the composition of their gut bacterial communities when compared with healthy controls (Table 1). However, these studies neither answer the cause or consequence question of dysbiosis in CRC, nor do they provide mechanistic insights by which the intestinal microbiota influences the development of CRC. Evidence for the association of human intestinal bacteria with CRC has stemmed from deep-sequencing technology, to date, provided by three independent studies that investigated microbial composition in healthy (off-tumor site) and late-stage CRC (on-tumor site) tissue.^45–47^ An enrichment of *Fusobacterium nucleatum* has been shown in CRC tissue, with a larger amount of *F. nucleatum* being associated with high degrees of microsatellite instability (MSI-high) and CpG island methylator phenotype (CIMP).^46–48^ Furthermore, *F. nucleatum* in colorectal carcinoma tissue was shown to be inversely proportional to the CD3^+^ T cell density, providing mechanistic evidence for the interactive roles of this microorganism in adaptive immunity,^49^ an important insight for the targeting of the microbiota and immunity in CRC prevention and therapy. A further validation for the connection between *F. nucleatum* and colon cancer, with a correlation to inflammatory factors, was provided by Wei et al.^50^ The same study also first reported a patient prognosis value of *B. fragilis* and *Fusobacterium prausnitzii* through the induction of intestinal inflammation, suggesting all three microorganisms as potential prognostic biomarkers for CRC.

**Table 1.** **Microbiota Bacteria Associated with Colorectal Cancer in Human Subjects**

Bacteria	Association	References
*Fusobacterium*	Enriched in human colon carcinoma	^45–47,50,87,88^
*Streptococcus bovis*	Increased prevalence in patients with carcinoma of the colon	^89^
*Clostridium septicum*	Aortic infections associated with colonic adenocarcinoma/polyps	^90^
*Slackia*	Overrepresented in tissue of CRC patients	^45^
*Collinsella*	Overrepresented in tissue of CRC patients	^45^
*Roseburia*	Overrepresented in tissue of CRC patients	^45^
*Faecalibacterium*	Overrepresented in tissue of CRC patients	^45^
*Bacteroides fragilis*	Increased prevalence of ETBF in colon cancer patients	^50,91^
*Enterococcus faecalis*	Significantly higher populations in colorectal cancer patients	^92^
*Escherichia coli*	Enhanced adhesion and invasion in colorectal cancer tumors	^23,24,93,94^

CRC, colorectal cancer; ETBF, enterotoxigenic *Bacteroides fragilis.*

The first high-resolution map of the colonic microbiota associated with human CRC showed that *Coriobacteria* were overrepresented, whereas potentially pathogenic *Enterobacteria* were underrepresented in patients.^45^ First experiments using Denaturing Gradient Gel Electrophoresis and Ribosomal Intergenic Spacer Analysis fingerprinting, indicated striking differences in microbial communities between tumor and off-tumor tissue. Subsequent FLX 454 titanium pyrosequencing revealed significantly altered community structures of the microbiota related to tumor vs. off-tumor sites at higher resolution. In these studies, CRC was consistently associated with overrepresentation of *Coriobacteridae*, especially of the genera *Slackia* and *Collinsella*, and underrepresentation of *Citrobacter*, *Shigella*, *Cronobacter*, *Kluyvera*, *Serratia*, and *Salmonella* spp. of the *Enterobacteriaceae* family. Shifts in microbial composition are often the result of dramatic physiological and metabolic alterations in the colonic microenvironment during tumorigenesis; these changes seem to benefit the rise of tumor-associated commensal-like bacteria with subsequent underrepresentation of Enterobacteria that might be linked to CRC pathogenesis.^51,52^ Some of these tumor-associated bacteria are major butyrate-producing microorganisms with potentially protective functions in CRC. In this regard, butyrate induces cell cycle arrest and increased apoptosis of cancer cells,^53^ but also serves as an energy source for neoplastic colonocytes.

## The Microbiota and CRC in Animal Models

Studies addressing the role of the gut microbial ecosystem in CRC development using animal models are compiled in Table 2. The characterization of the gut microbiota in a murine model of AOM DSS-induced CRC has shown an enrichment in operational taxonomic units (OTUs) affiliated with members of the genera *Bacteroides*, *Odoribacter*, and *Akkermansia,* whereas OTUs affiliated with members of the *Prevotellaceae* and *Porphyromonadaceae* families were decreased.^54^ Furthermore, the administration of antibiotics in this model resulted in a drastic reduction in tumor size and number, implying that changes in the microbiota directly contribute to tumorigenesis.^54^ In contrast to conventionally raised mice, germ-free glutathione peroxidase double knockout (GPX-DKO) mice as well as interleukin-10-deficient (IL10^−/−^) mice, treated with AOM, display normal colon histology and no tumor development.^30,33^ Furthermore, in the Apc^Min/+^ murine model of colon carcinogenesis, germ-free housing showed a reduction in tumor burden, and the introduction of *B. fragilis* or *F. nucleatum* increased carcinogenesis.^26,55,56^ These results provide *in vivo* evidence for the influence of bacteria on carcinogenesis.

**Table 2.** **Microbiota Bacteria Associated with Colorectal Cancer in Murine Models**

Bacteria	Association	References
*Bacteroides fragilis*	Enterotoxigenic *B. fragilis* (ETBF) augments spontaneous colon cancer in multiple intestinal neoplasia (Min) mice	^26,91^
*Bacteroides vulgatus*	Monoassociation of AOM-IL10^−/−^ mice caused mild colorectal tumorigenesis	^30^
*Bifidobacterium longum*	Decreased the incidence of AOM-induced large aberrant crypt foci, which are predictive of tumor incidence, and IQ-induced colon tumors and multiplicity	^73,81^
*Citrobacter rodentium & Citrobacter freundii*	Induces colonic crypt hyperplasia and increases the susceptibility to neoplastic transformation in mice; reduces latent period for appearance of 1,2-dimethylhydrazine (DMH) tumors	^31,92^
*Enterococcus faecalis*	Triggers adenocarcinoma in IL-10 KO mice	^21,93^
*Escherichia coli*	*E. coli* NC101 promotes invasive carcinoma in AOM-IL10^−/−^ mice; *E. coli* 11G5 increases colonic polyps in multiple intestinal neoplasia (Min) mice.	^23,94,95^
*Helicobacter hepaticus*	Promotes colon tumorigenesis in the BALB-RagMin (C.Cg-Rag2^−/−^ApcMin^−/−^) mouse, and in the Smad3^−/−^ mouse	^96,97^
*H. hepaticus & Helicobacter bilis*	Induction of colon cancer through dual infection in Mdr1a^−/−^ mice	^97^
*Helicobacter typhlonius & Helicobacter rodentium*	Coinfection increases incidence of inflammation-associated colon neoplasia in IL10^−/−^ mice	^98^

Nucleotide-binding oligomerization domain-containing protein 2 (NOD2) is a cytoplasmic pattern recognition receptor that is linked to the development of Crohn's disease in humans. Dysbiotic microbiota in NOD2^−/−^ mice contributed to the development of colitis and colitis-associated cancer (CAC).^57^ In addition, disease risk was ameliorated in NOD2^−/−^ mice after the treatment with antibiotics or anti-IL-6 receptor-neutralizing antibodies. Most interestingly, the transfer of dysbiotic microbiota into germ-free wild-type (WT) mice again caused the development of colitis and CAC. Likewise, transplanting the normal microbiota from WT mice into NOD2^−/−^ mice reduced disease risk.^57^ These findings exemplify a role of microbial communities in inflammation and carcinogenesis. Furthermore, these observations propose that the manipulation of a dysbiotic microbiota could offer a possible therapeutic approach in the treatment of CRC and other human intestinal diseases.

## The Bacterial Driver-Passenger Model

In light of potentially distinct functions of bacterial groups in colonic tumorigenesis, a bacterial driver–passenger model for CRC was proposed.^58^ Bacterial drivers are defined as intestinal bacteria with pro-carcinogenic features that may initiate CRC development. For instance, a pro-carcinogenic feature of particular *E. coli* strains, harboring the genotoxin colibactin, can induce single-strand DNA breaks, and thereby increase the mutation rate of infected cells.^22^ Another bacterial CRC driver was identified in a mouse model of ETBF-induced colitis and carcinogenesis. In this model, ETBF can enhance tumorigenesis, possibly through the induction of a persistent TH17-type inflammatory response, causing DNA damage and genetic instability in human cells.^26,59^ In humans, potentially pathogenic Enterobacteria, such as *Shigella* spp., are rare in healthy individuals, but are overrepresented in nonmalignant colonic mucosa of patients with adenomas.^60,61^ This finding supports the early CRC-stage association of such species with the intestinal mucosa, suggesting a role for bacterial drivers.

In contrast, intestinal bacterial passengers constitute relatively poor colonizers of a healthy colon that have a competitive advantage in the tumor microenvironment and, therefore, outcompete bacterial drivers of CRC. For example, the distorted colon wall structure in a tumor microenvironment may expose the collagen fibers in the basement membrane, allowing access to bacteria such as *S. gallolyticus* subsp. *gallolyticus*.^62^ Accordingly, the prevalence of *S. gallolyticus* subsp. *gallolyticus* in the general population is much lower than that found in patients with colonic adenomas and CRC samples,^45–47,63^ suggesting that these microorganisms represent bacterial passengers. Another example of potential bacterial passengers are the *Fusobacterium* spp., which are found consistently overrepresented in tumor samples^45–47^ with no clear role in CRC development and progression. This would support the idea for a role of *Fusobacterium* spp. as passenger bacteria.

While the driver–passenger model does not exclude passenger bacteria as active culprits of tumor progression, it rather suggests that their involvement may be in later disease stages. Nevertheless, the composition of the indigenous rather than the tumor microbiota of patients with CRC would be a more relevant indicator for the risk of developing colon cancer. An increased understanding of shifts in the microbiota would enable the identification of bacterial drivers of colon cancer and thereby provide an invaluable tool for early diagnosis of colon cancer and new prevention strategies.

## Therapeutic Implications

Taking into consideration that microbe–host interactions contribute to tumorigenesis, several different strategies have been evaluated in the context of CRC prevention. In this regard, bacteria-induced ROS production and its consequent DNA damage is one possible target for antineoplastic chemoprevention in CRC. Treatment with an inhibitor of polyamine catabolism has been shown to decrease proliferation and tumorigenesis in ETBF-induced mouse models and Apc^Min/+^ rodents.^25^ Another mechanism by which the colonic microbiota might have a role in CRC antineoplastic strategies relies on microbial fermentation products. Butyrate, a short-chain fatty acid (SCFA) produced during microbial fermentation of indigestible complex carbohydrates such as fiber, for instance, initiates growth arrest and apoptosis of colonic epithelial cells *in vitro*.^64^ SCFA might not only have antineoplastic functions, but also an important anti-inflammatory role, targeting G-protein-coupled receptor 43 (GPR43).^65^

Several studies have highlighted the importance of the microbiota composition in the tumor patient response to chemotherapy or checkpoint blockade immunotherapy.^66,67^ Of importance here are findings of Sivan et al. (2015) and Vétizou et al. (2015), demonstrating that constituents of the intestinal microbiota can influence the outcome of tumor immunotherapy through the augmentation of dendritic cell activation and subsequent priming of antitumor T cell responses.^68,69^ In light of the heterogeneous antitumor immunity of patients, the identification of microbes that may serve as biomarkers for predicting therapeutic responses as well as maximizing the benefit of clinical cancer immunotherapy, is an obvious growing field of research.

Mechanistically, microbes may promote carcinogenesis by different processes, such as toxic metabolite production and genotoxic biosynthesis,^70^ providing a further CRC treatment approach. A recent study aimed at inhibiting toxic effects of colibactin toxin-producing *E. coli*, which represent frequent colonizers of CRCs. Two boronic acid-based compounds were identified, which were shown to bind to the active site of the ClbP enzyme involved in the synthesis of colibactin, and shown to suppress DNA damage and tumorigenesis induced by *pks*-harboring bacteria.^71^ While confirming the importance of colibactin toxin-producing *E. coli* in colon tumorigenesis, this study also provides a novel family of inhibitors to target *pks*-harboring bacteria in the treatment of CRC.

The consumption of lactic acid bacteria, together with some dietary factors, such as fibers and cruciferous vegetables, has been found to be inversely correlated with the incidence of CRC in humans.^72^ These bacteria have been found to inhibit cancer development in culture^73–76^ and in animal models of CRC.^77–81^ In the case of the mutagenic compounds known as heterocyclic amines (HCAs), lactic acid-producing bacteria can prevent HCA-related induction of DNA damage by direct binding to these amines through the components of their cell wall.^72^ The presence of this specific population of bacteria can be enhanced by their direct ingestion (probiotics), usually in fermented dairy products, or by the consumption of nondigestible oligosaccharides (prebiotics), which can act as specific substrates for lactobacilli and Bifidobacteria.^73^ Importantly, two of these nondigestible prebiotics, lactulose and inulin, have also been shown to decrease the level of carcinogen-induced DNA damage in the colon of rodents.^73,82^ The modulation of the gut microbiota by probiotics and prebiotics may positively impact on the crosstalk between the immune system and the microbiota. Preclinical models provide evidence that the administration of probiotics has protective effects against CRC by antineoplastic and antiproliferative activities, reduction in aberrant crypt foci, SCFA formation, downregulation of proinflammatory cytokines, inhibition of pathogens and cancer-causing microbes, immunostimulation, and reduction of pro-carcinogenic enzymatic activity.^83^

## Conclusion

In animal models, environmental and dietary factors, including the intestinal microbiota, seem to play a critical role in the early stages of CRC formation. Even though no direct link between the colonic microbiota and the initiation of intestinal tumorigenesis in humans has yet been established, a growing body of evidence suggests that the selection of genotoxic bacteria might play an important role in CRC initiation and promotion. Alterations in the microbiota composition and function that were thought to be a passive reaction to changes in the microenvironment might in fact be an active contributing factor to the development of CRC. The notion of a causal link between dysbiosis and CRC opens a field of microbial genes as potential biomarkers for CRC.^84–86^ An increased understanding of bacterial community shifts taking place in the context of CRC, will allow for future therapeutic and preventive strategies, based on intestinal microbiota modulation and microbe–host interactions, which may form a crucial part of the armamentarium against this lethal type of cancer.

## Funding

This work was supported by the German Research Foundation (DFG) and the Technische Universität München within the Open Access Publishing Funding Program.

## Abbreviations Used

AOM: azoxymethane
BFT: Bacteroides fragilis toxin
CAC: colitis-associated cancer
CRC: colorectal cancer
DSS: dextran sodium sulfate
ETBF: enterotoxigenic Bacteroides fragilis
HBV: hepatitis B virus
HCA: heterocyclic amine
NF-κB: nuclear factor-κB
OUT: operational taxonomic units
ROS: reactive oxygen species
SCFA: short-chain fatty acid
SMO: spermine oxidase
WT: wild type
